# Supplementary material for: How to calibrate Gaussian two-factor model using swaption
Source: PLoS One. 2023 Feb 23;18(2):e0280829. doi: 10.1371/journal.pone.0280829 (PMC9949672; doi:10.1371/journal.pone.0280829)
Supplement: S1 Appendix — (DOCX) [file pone.0280829.s001.docx]

**S1 Appendix: Model Description**

**Model dynamics**

We assume that $a\left( t \right)$, $b\left( t \right)$, $\sigma\left( t \right)$, and $\eta\left( t \right)$ are piecewise constant over $\left[ t_{0},t_{1},\cdots\right]$. The parameters are defined such that $a\left( t \right)=a_{i}$, where $t\in\left[ t_{i},t_{i+1} \right)$, and similarly for other parameters. The solutions for $x\left( t \right)$ and $y\left( t \right)$ given the filtration $F_{s}$ are as follows:

|  | $x(t)\vert F_{s}=\frac{1}{A\left( t \right)}[x\left( s \right)A\left( s \right)+\int_{s}^{t} A\left( u \right)\sigma\left( u \right)dW_{1}\left( u \right)]\text{,}$ $y(t)\vert F_{s}=\frac{1}{B\left( t \right)}[y\left( s \right)B\left( s \right)+\int_{s}^{t} B\left( u \right)\eta\left( t \right)dW_{2}\left( u \right)]\text{.}$ | (1) |
| --- | --- | --- |

The functions $A\left( t \right)$ and $B(t)$ are

|  | $A\left( t \right)=exp\left( \int_{0}^{t} a\left( u \right)du \right) and B\left( t \right)=\text{exp}\left( \int_{0}^{t} b\left( u \right)du \right)\text{.}$ | (2) |
| --- | --- | --- |

**Moments of** $\boldsymbol{x}\left( \boldsymbol{t} \right)$ **and** $\boldsymbol{y}\left( \boldsymbol{t} \right)$

Assume $t_{s}<\underline{t}<t_{s+1}$ and $t_{e}<t<t_{e+1}$.

The mean of $x\left( t \right)\text{ and }y\left( t \right)$are as follows:

|  | $E\left[ x\left( t \right) \vert F_{\underline{t}} \right]=\text{exp}\left( -\int_{\underline{t}}^{t} a\left( u \right)du \right)x\left( \underline{t} \right)$ $=\text{exp}\left( -a_{s}\left( t_{s+1}-\underline{t} \right)-\sum_{i=s+1}^{e-1} a_{i}\left( t_{i+1}-t_{i} \right)-a_{e}\left( t-t_{e} \right) \right)x\left( \underline{t} \right)\text{,}$ $E\left[ y\left( t \right) \vert F_{\underline{t}} \right]=\text{exp}\left( -\int_{\underline{t}}^{t} b\left( u \right)du \right)y\left( \underline{t} \right)$ $=\text{exp}\left( -b_{s}\left( t_{s+1}-\underline{t} \right)-\sum_{i=s+1}^{e-1} b_{i}\left( t_{i+1}-t_{i} \right)-b_{e}\left( t-t_{e} \right) \right)y\left( \underline{t} \right)\text{.}$ | (3) |
| --- | --- | --- |

The variances of $x\left( t \right)\text{ and }y\left( t \right)$are as follows.

|  | $Var\left( x\left( t \right) \vert F_{\underline{t}} \right)=\int_{\underline{t}}^{t} \sigma^{2}\left( u \right)\text{exp}\left( -2\int_{\underline{t}}^{u} a\left( s \right)d\left( s \right)ds \right)du$ $=\int_{\underline{t}}^{t_{s+1}} \sigma_{s}^{2}e^{-2a_{s}\left( u-\underline{t} \right)}du+\sum_{i=s+1}^{e-1} \sigma_{i}^{2}\int_{t_{i}}^{t_{i=1}} \text{exp}\left( -2\left( a_{s}\left( t_{s+1}-\underline{t} \right)+\sum_{j=s+1}^{i-1} a_{j}\left( t_{j+1}-t_{j} \right)+a_{i}\left( u-t_{i} \right) \right) \right)du+\sigma_{e}^{2}\int_{t_{e}}^{t} \text{exp}\left( -2\left( a_{s}\left( t_{s+1}-\underline{t} \right)+\sum_{j=s+1}^{e-1} a_{j}\left( t_{j+1}-t_{j} \right)+a_{e}\left( u-t_{e} \right) \right) \right)du$ $=\frac{\sigma_{s}^{2}}{2a_{s}}\left( 1-e^{-2a_{s}\left( t_{s+1}-\underline{t} \right)} \right)+\sum_{i=s+1}^{e-1} \frac{\sigma_{i}^{2}}{2a_{i}}\text{exp}\left( -2\left( a_{s}\left( t_{s+1}-\underline{t} \right)+\sum_{j=s+1}^{i-1} a_{j}\left( t_{j+1}-t_{j} \right) \right) \right)\left( 1-e^{-2a_{i}\left( t_{i+1}-t_{i} \right)} \right)+\frac{\sigma_{e}^{2}}{2a_{e}}\text{exp}\left( -2\left( a_{s}\left( t_{s+1}-\underline{t} \right)+\sum_{j=s+1}^{e-1} a_{j}\left( t_{j+1}-t_{j} \right) \right) \right)\left( 1-e^{-2a_{e}\left( t_{i+1}-t_{e} \right)} \right)\text{.}$ | (4) |
| --- | --- | --- |

For the $Var\left( y\left( t \right) | F_{\underline{t}} \right)$, we can replace $\sigma^{'}s$ by $\eta$, and $a^{'}s$ by $b$.

Finally, we can get covariance of $x\left( t \right),y\left( t \right)$.

|  | $Cov\left( x\left( t \right),y\left( t \right) \vert F_{\underline{t}} \right)=\rho\int_{\underline{t}}^{t} \sigma\left( u \right)\eta\left( u \right)e^{\left( -\int_{\underline{t}}^{u} \left( a\left( s \right)+b\left( s \right) \right)ds \right)}du$ $=\frac{\rho\sigma_{s}\eta_{s}}{a_{s}+b_{s}}\left( 1-e^{-\left( a_{s}+b_{s} \right)\left( t_{s+1}-\underline{t} \right)} \right)+\sum_{i=s+1}^{e-1} \frac{\rho\sigma_{i}\eta_{i}}{a_{i}+b_{i}}e^{\left( -\left( a_{s}+b_{s} \right)\left( t_{s+1}-\underline{t} \right)-\sum_{j=s+1}^{i-1} \left( a_{j}+b_{j} \right)\left( t_{j+1}-t_{j} \right) \right)}\left( 1-e^{-\left( a_{i}+b_{i} \right)\left( t_{i+1}-t_{i} \right)} \right)+\frac{\rho\sigma_{e}\eta_{e}}{a_{e}+b_{e}}e^{\left( -\left( a_{s}+b_{s} \right)\left( t_{s+1}-\underline{t} \right)-\sum_{j=s+1}^{e-1} \left( a_{j}+b_{j} \right)\left( t_{j+1}-t_{j} \right) \right)}\left( 1-e^{-\left( a_{e}+b_{e} \right)\left( t-t_{e} \right)} \right)\text{.}$ | (5) |
| --- | --- | --- |

Then, the variance of $x\left( t \right)+y\left( t \right)$ is defined as:

|  | $Var\left( x\left( t \right)+y\left( t \right) \vert F_{\underline{t}} \right)=Var\left( x\left( t \right) \vert F_{\underline{t}} \right)+Var\left( y\left( t \right) \vert F_{\underline{t}} \right)+2Cov\left( x\left( t \right),y\left( t \right) \vert F_{\underline{t}} \right)\text{.}$ | (6) |
| --- | --- | --- |

**Moments of** $\int\left( \boldsymbol{x}\left( \boldsymbol{u} \right)\mathbf{+}\boldsymbol{y}\left( \boldsymbol{u} \right) \right)\boldsymbol{du}$

The mean can be calculated as follows:

|  | $\frac{1}{x\left( t \right)}E\left[ \int_{t}^{T} x\left( u \right)du \vert F_{t} \right]$ $=\int_{t}^{t_{s+1}} \frac{A\left( t \right)}{A\left( u \right)}du+\sum_{i=s+1}^{e-1} \int_{t_{i}}^{t_{i+1}} \frac{A\left( t \right)}{A\left( u \right)}du+\int_{T}^{t_{e}} \frac{A\left( t \right)}{A\left( u \right)}du$ $=\int_{t}^{t_{s+1}} e^{-a_{s}\left( u-t \right)}du+\sum_{i=s+1}^{e-1} \int_{t_{i}}^{t_{i+1}} e^{-a_{s}\left( t_{s+1}-t \right)-\sum_{j=s+1}^{i-1} a_{j}\left( t_{j+1}-t_{j} \right)-a_{i}\left( u-t_{i} \right)}du+\int_{T}^{t_{e}} e^{-a_{s}\left( t_{s+1}-t \right)-\sum_{j=s+1}^{e-1} a_{j}\left( t_{j+1}-t_{j} \right)-a_{e}\left( u-t_{e} \right)}du$ $=-\frac{e^{-a_{s}\left( t_{s+1}-t \right)}-1}{a_{s}}+e^{-a_{s}\left( t_{s+1}-t \right)}\sum_{i=s+1}^{e-1} \left( -\frac{1}{a_{i}} \right)e^{-\sum_{j=s+1}^{i-1} a_{j}\left( t_{j+1}-t_{j} \right)}\left( e^{-a_{i}\left( t_{i+1}-t_{i} \right)}-1 \right)+e^{-a_{s}\left( t_{s+1}-t \right)}e^{-\sum_{j=s+1}^{e-1} a_{j}\left( t_{j+1}-t_{j} \right)}\left( -\frac{1}{a_{e}} \right)\left( e^{-a_{e}\left( T-t_{e} \right)}-1 \right)$ $=\frac{1}{a_{s}}-e^{-a_{s}\left( t_{s+1}-u \right)}\left[ \frac{1}{a_{s}}-\sum_{i=s+1}^{e-1} a_{i}^{-1}e^{-\sum_{j=s+1}^{i-1} a_{j}\left( t_{j+1}-t_{j} \right)}\left( 1-e^{-a_{i}\left( t_{i+1}-t_{i} \right)} \right)-a_{e}^{-1}e^{-\sum_{j=s+1}^{e-1} a_{j}\left( t_{j+1}-t_{j} \right)}\left( 1-e^{-a_{e}\left( T-t_{e} \right)} \right) \right]\text{.}$ | (7) |
| --- | --- | --- |

For notational convenience, we define the piecewise constant $C_{x}\left( T;t_{s},t_{e} \right)$ such that

|  | $\frac{1}{x\left( t \right)}E\left[ \int_{t}^{T} x\left( u \right)du \vert F_{t} \right]=\frac{1}{a_{s}}-e^{-a_{s}\left( t_{s+1}-u \right)}C_{x}\left( T;t_{s},t_{e} \right)\text{.}$ | (8) |
| --- | --- | --- |

Note: if $a$ = constant, $C_{x}=\frac{1}{a}e^{-a\left( T-t_{s+1} \right)}$.

Multiplying both sides by $x\left( t \right)$ yields the solution. In addition, $E\left[ \int_{t}^{T} y\left( u \right)du | F_{t} \right]$ and $C_{y}\left( T;t_{s},t_{e} \right)$ can be defined similarly.

For the variance, we will calculate $\int_{t}^{T} x\left( t \right)du$.

|  | $\int_{t}^{T} x\left( u \right)du=Tx\left( T \right)-tx\left( t \right)-\int_{t}^{T} udx\left( u \right)$ $=\left( T-t \right)x\left( t \right)+\int_{t}^{T} \left( T-u \right)dx\left( u \right)$ $=\left( T-t \right)x\left( t \right)+\int_{t}^{T} \left( T-u \right)\left( -a\left( u \right)x\left( u \right)du+\sigma\left( u \right)dW_{u}^{1} \right)$ $=\left( T-t \right)x\left( t \right)+\int_{t}^{T} \left( T-u \right)\sigma\left( u \right)dW_{u}^{1}-\int_{t}^{T} \left( T-u \right)a\left( u \right)x\left( u \right)du$ $=\left( T-t \right)x\left( t \right)+\int_{t}^{T} \left( T-u \right)\sigma\left( u \right)dW_{u}^{1}+-\int_{t}^{T} \left( T-u \right)a\left( u \right)\frac{1}{A\left( u \right)}\left( A\left( t \right)x\left( t \right)+\int_{t}^{u} A\left( s \right)\sigma\left( s \right)dW_{s}^{1}ds \right)du$ $=\left( T-t \right)x\left( t \right)-x\left( t \right)\int_{t}^{T} \left( T-u \right)a\left( u \right)\frac{A\left( t \right)}{A\left( u \right)}du++\int_{t}^{T} \left( T-u \right)\sigma\left( u \right)dW_{u}^{1}-\underset{\text{Last Term}}{\underbrace{\int_{t}^{T} \left( T-u \right)a\left( u \right)\left( \int_{t}^{u} \frac{A\left( s \right)}{A\left( u \right)}\sigma\left( s \right)dW_{s}^{1}ds \right)du}}\text{.}$ | (9) |
| --- | --- | --- |

The last two terms are the volatility terms for $\int_{t}^{T} x\left( u \right)du$.

Note: $\frac{1}{x\left( t \right)}E\left[ \int_{t}^{T} x\left( u \right)du | F_{t} \right]=\left( T-t \right)-\int_{t}^{T} \left( T-u \right)a\left( u \right)\frac{A\left( t \right)}{A\left( u \right)}du$.

We can rewrite the last term as follows:

|  | $\text{Last term}=\int_{t}^{T} a\left( u \right)\left( T-u \right)A^{-1}\left( u \right)\left( \int_{t}^{u} A\left( s \right)\sigma\left( s \right)dW_{s}^{1}ds \right)du$ $=\int_{t}^{T} \left( \int_{t}^{u} A\left( s \right)\sigma\left( s \right)dW_{s}^{1} \right)\frac{\partial}{\partial u}\left( \int_{t}^{u} a\left( v \right)\left( T-v \right)A^{-1}\left( v \right)dv \right)du$ $=\left( \int_{t}^{T} A\left( s \right)\sigma\left( s \right)dW_{s}^{1} \right)\left( \int_{t}^{T} a\left( v \right)\left( T-v \right)A^{-1}\left( v \right)dv \right)-\int_{t}^{T} \left( \int_{t}^{u} a\left( v \right)\left( T-v \right)A^{-1}\left( v \right)dv \right)A\left( u \right)\sigma\left( u \right)dW_{u}^{1}$ $=\int_{t}^{T} \left( \int_{u}^{T} a\left( v \right)\left( T-v \right)A^{-1}\left( v \right)dv \right)A\left( u \right)\sigma\left( u \right)dW_{u}^{1}$ $=\int_{t}^{T} \sigma\left( u \right)\left( \left( T-u \right)-\frac{1}{x\left( u \right)}E\left[ \int_{u}^{T} x\left( s \right)ds \vert F_{u} \right] \right)dW_{u}^{1}\text{.}$ | (10) |
| --- | --- | --- |

Substituting the last term into the equation for $\int_{t}^{T} x\left( u \right)du$, and defining $V_{x}\left( u,T \right)$ as follows:

|  | $\text{Volatility term}=\int_{t}^{T} \sigma\left( u \right)\frac{1}{x\left( u \right)}E\left[ \int_{u}^{T} x\left( s \right)ds \vert F_{u} \right]dW_{u}^{1}\text{.}$ | (11) |
| --- | --- | --- |

Then, the variance of $\int_{t}^{T} x\left( u \right)du$ is

|  | $Var\left( \int_{t}^{T} x\left( u \right)du \right)=\int_{t}^{T} \frac{1}{\sigma\left( u \right)^{2}}\left\{ \frac{1}{x\left( u \right)^{2}}E\left[ \int_{u}^{T} x\left( s \right)ds \vert F_{u} \right] \right\}^{2}du$ $={\int_{t}^{t_{s+1}} \sigma_{s}^{2}\left[ \frac{1}{a_{s}}-e^{-a_{s}\left( t_{s+1}-u \right)}C_{x}\left( T;t_{s},t_{e} \right) \right]}^{2}du+\sum_{i=s+1}^{e-1} \int_{t_{i}}^{t_{i+1}} \sigma_{i}^{2}\left[ \frac{1}{a_{i}}-e^{-a_{i}\left( t_{i+1}-u \right)}C_{x}\left( T;t_{i},t_{e} \right) \right]^{2}du+\int_{t_{e}}^{T} \frac{\sigma_{e}^{2}}{a_{e}^{2}}\left[ 1-e^{-a_{e}\left( T-u \right)} \right]^{2}du\text{.}$ | (12) |
| --- | --- | --- |

(The last term collapses to the constant volatility case over $\left[ t_{e},T \right]$)

For notational ease, define $V_{x}^{'}\left( \underline{t},\overline{t},T;i,t_{e} \right)$ as follows: ($t_{i}<\underline{t}<\overline{t}<t_{i+1}$)

|  | $V_{x}^{'}\left( \underline{t},\overline{t},T;i,t_{e} \right)=\int_{\underline{t}}^{\overline{t}} \sigma_{i}^{2}\left[ \frac{1}{a_{i}}-e^{-a_{i}\left( t_{i+1}-u \right)}C_{x}\left( T;t_{i},t_{e} \right) \right]^{2}du$ $=\int_{\underline{t}}^{\overline{t}} \sigma_{i}^{2}\left[ \frac{1}{a_{i}^{2}}-\frac{2}{a_{i}}C_{x}\left( T;t_{i},t_{e} \right)e^{-a_{i}t_{i+1}+a_{i}u}+C_{x}\left( T;t_{i},t_{e} \right)^{2}e^{-2a_{i}t_{i+1}+2a_{i}u} \right]du$ $=\sigma_{i}^{2}\left[ \frac{\overline{t}-\underline{t}}{a_{i}^{2}}-\frac{2}{a_{i}^{2}}C_{x}\left( T;t_{i},t_{e} \right)e^{-a_{i}t_{i+1}}\left( e^{a_{i}\overline{t}}-e^{a_{i}\underline{t}} \right)+\frac{C_{x}\left( T;t_{i},t_{e} \right)^{2}}{2a_{i}}e^{-2a_{i}t_{i+1}}\left( e^{2a_{i}\overline{t}}-e^{2a_{i}\underline{t}} \right) \right]\text{.}$ | (13) |
| --- | --- | --- |

Then, the variance of $\int_{t}^{T} x\left( u \right)du$ is

|  | $Var\left( \int_{t}^{T} x\left( u \right)du \vert F_{t} \right)=V_{x}^{'}\left( t,t_{s+1},T;s,t_{e} \right)+\sum_{i=s+1}^{e-1} V_{x}^{'}\left( t_{i},t_{i+1},T;i,t_{e} \right)+\frac{\sigma_{e}^{2}}{a_{e}^{2}}\left[ T-t_{e}+\frac{2}{a_{e}}e^{-a_{e}\left( T-t_{e} \right)}-\frac{1}{2a_{e}}e^{-2a_{e}\left( T-t_{e} \right)}-\frac{3}{2a_{e}} \right]\text{.}$ | (14) |
| --- | --- | --- |

Similarly, we can define $Var\left( \int_{t}^{T} y\left( u \right)du | F_{t} \right)$.

For the covariance term, define $V_{xy}^{'}\left( \underline{t},\overline{t},T;i,t_{e} \right)$ such that,

|  | $V_{xy}^{'}\left( \underline{t},\overline{t},T;i,t_{e} \right)=\rho\int_{\underline{t}}^{\overline{t}} \sigma_{i}\eta_{i}\left[ \frac{1}{a_{i}}-e^{-a_{i}\left( t_{i+1}-u \right)}C_{x}\left( T;t_{m},t_{e} \right) \right]\left[ \frac{1}{b_{i}}-e^{-b_{i}\left( t_{i+1}-u \right)}C_{y}\left( T;t_{m},t_{e} \right) \right]du$ $=\rho\sigma_{i}\eta_{i}\left[ \frac{\overline{t}-\underline{t}}{a_{i}b_{i}}-\frac{C_{x}\left( T;t_{i},t_{e} \right)}{a_{i}b_{i}}\left( e^{-a_{i}\left( t_{i+1}-\overline{t} \right)}-e^{-a_{i}\left( t_{i+1}-\underline{t} \right)} \right)-\frac{C_{y}\left( T;t_{i},t_{e} \right)}{a_{i}b_{i}}\left( e^{-b_{i}\left( t_{i+1}-\overline{t} \right)}-e^{-b_{i}\left( t_{i+1}-\underline{t} \right)} \right)+\frac{C_{x}\left( T;t_{i},t_{e} \right)C_{y}\left( T;t_{i},t_{e} \right)}{a_{i}+b_{i}}e^{-\left( a_{i}+b_{i} \right)t_{i+1}}\left( e^{\left( a_{i}+b_{i} \right)\overline{t}}-e^{\left( a_{i}+b_{i} \right)\underline{t}} \right) \right]$ | (15) |
| --- | --- | --- |

Thus, the covariance term is defined as:

|  | $cov\left( \int_{t}^{T} x\left( u \right)du,\int_{t}^{T} y\left( u \right)du \vert F_{t} \right)=V_{xy}^{'}\left( t,t_{s+1},T;s,t_{e} \right)+\sum_{i=s+1}^{e-1} V_{xy}^{'}\left( t_{i},t_{i+1},T;i,t_{e} \right)++2\rho\frac{\sigma_{e}\eta_{e}}{a_{e}b_{e}}\left[ T-t_{e}+\frac{e^{a_{e}\left( T-t_{e} \right)}-1}{a_{e}}+\frac{e^{-b_{e}\left( T-t_{e} \right)}-1}{b_{e}}-\frac{e^{-\left( a_{e}+b_{e} \right)\left( T-t_{e} \right)}-1}{a_{e}+b_{e}} \right]\text{.}$ | (16) |
| --- | --- | --- |

Finally,

|  | $Var\left( \int_{t}^{T} \left( x\left( u \right)+y\left( u \right) \right)du \vert F_{t} \right)$ $=Var\left( \int_{t}^{T} x\left( u \right)du \vert F_{t} \right)+Var\left( \int_{t}^{T} y\left( u \right)du \vert F_{t} \right)+2cov\left( \int_{t}^{T} x\left( u \right)du,\int_{t}^{T} y\left( u \right)du \vert F_{t} \right)\text{.}$ | (17) |
| --- | --- | --- |

**Zero-coupon bond price formula**

For notation convenience, we will define as:

|  | $M\left( t,T \right)\triangleq E\left[ \int_{t}^{T} \left( x\left( u \right)+y\left( u \right) \right)du \vert F_{t} \right]\text{,}$ $V\left( t,T \right)\triangleq Var\left[ \int_{t}^{T} \left( x\left( u \right)+y\left( u \right) \right)du \vert F_{t} \right]\text{.}$ | (18) |
| --- | --- | --- |

Thus, the zero-coupon bond price can be written as:

|  | $P\left( t,T \right)=E\left[ \text{exp}\left( -\int_{t}^{T} r_{s} ds \right) \vert F_{t} \right]$ $=E\left[ \text{exp}\left( -\int_{t}^{T} \phi\left( u \right)du \right)\text{exp}\left( -\int_{t}^{T} \left( x\left( s \right)+y\left( s \right) \right)ds \right) \vert F_{t} \right]$ $=\text{exp}\left( -\int_{t}^{T} \phi\left( u \right)du \right)\text{exp}\left( -M\left( t,T \right)+\frac{1}{2}V\left( t,T \right) \right)\text{.}$ | (19) |
| --- | --- | --- |

Then,

|  | $P^{M}\left( 0,T \right)=e^{\left( -\int_{0}^{T} \phi\left( u \right)du+\frac{1}{2}V\left( 0,T \right) \right)}\to e^{\left( -\int_{0}^{T} \phi\left( u \right)du \right)}=P^{M}\left( 0,T \right)e^{\left( -\frac{1}{2}V\left( 0,T \right) \right)}\text{,}$ $e^{\left( -\int_{t}^{T} \phi\left( u \right)du \right)}=e^{\left( -\int_{0}^{T} \phi\left( u \right)du \right)}/e^{\left( -\int_{0}^{t} \phi\left( u \right)du \right)}=\frac{P^{M}\left( 0,T \right)e^{-\frac{1}{2}V\left( 0,T \right)}}{P^{M}\left( 0,t \right)e^{-\frac{1}{2}V\left( 0,t \right)}}\text{.}$ | (20) |
| --- | --- | --- |

Therefore, the bond price is

|  | $P\left( t,T \right)=\frac{P^{M}\left( 0,T \right)}{P^{M}\left( 0,t \right)}e^{\frac{1}{2}\left( V\left( t,T \right)-V\left( 0,T \right)+V\left( 0,t \right) \right)-M\left( t,T \right)}$ $\mathcal{= A}\left( t,T \right)exp\left\{ \mathcal{-B}\left( a,t,T \right)x\left( t \right)\mathcal{-B}\left( b,t,T \right)y\left( t \right) \right\}\text{,}$ | (21) |
| --- | --- | --- |

where

$$\mathcal{A}\left( t,T \right)=exp\left\{ \frac{1}{2}V_{P}^{2}\left( t,T \right)-\int_{t}^{T} \Phi\left( u \right)du \right\}\text{,}$$

$$\mathcal{B}\left( a,t,T \right)=\frac{1-e^{-a\left( T-t \right)}}{a}\text{,}$$

$$\mathcal{B}\left( b,t,T \right)=\frac{1-e^{b\left( T-t \right)}}{b}\text{.}$$

**An approximation of the swaption normal volatility**

We assume that $a, b, \sigma, \eta,$ and $\rho$ are constants. The swaption payoff can be expressed as

|  | $V_{swaption}\left( T_{0} \right)=A\left( T_{0} \right)\left( S\left( T_{0} \right)-c \right)^{+}\text{,}$ | (22) |
| --- | --- | --- |

where $A\left( t \right)$ and $S\left( t \right)$ are the swap annuity and forward swap rate, respectively:

|  | $A\left( t \right)\triangleq A_{0,N}\left( t \right)=\sum_{i=0}^{N-1} \tau_{i}P\left( t,T_{i+1} \right)\text{,}$ $S\left( t \right)\triangleq S_{0,N}\left( t \right)=\frac{P\left( t,T_{0} \right)-P\left( t,T_{N} \right)}{A\left( t \right)}\text{.}$ | (23) |
| --- | --- | --- |

Let $Q^{A}$ be the measure induced by using $A\left( t \right)$ as the numeraire, such that

|  | $V_{swaption}\left( 0 \right)=A\left( 0 \right)E^{A}\left( \left( S\left( T_{0} \right)-c \right)^{+} \right)\text{,}$ | (24) |
| --- | --- | --- |

where $E^{A}$ denotes expectation in measure $Q^{A}$.

By the Bachelier formula, swap rate dynamics is

|  | $dS\left( t \right)=\nu\left( t \right)dW^{A}\left( t \right)\text{.}$ | (25) |
| --- | --- | --- |

We know that $S\left( t \right)$ and $A\left( t \right)$ must be deterministic functions of $x\left( t \right),y\left( t \right):$

|  | $S\left( t \right)=S\left( t,x\left( t \right),y\left( t \right) \right)\text{,} A\left( t \right)=A\left( t,x\left( t \right),y\left( y \right) \right)\text{.}$ | (26) |
| --- | --- | --- |

Therefore, from Ito's lemma,

|  | $dS\left( t \right)$ $=\frac{\partial S}{\partial x}\left( t,x\left( t \right),y\left( t \right) \right)dx\left( t \right)+\frac{\partial S}{\partial y}\left( t,x\left( t \right),y\left( t \right) \right)dy\left( t \right)+\frac{\partial^{2}S}{\partial x\partial y}\left( t,x\left( t \right),y\left( t \right) \right)dx\left( t \right)dy\left( t \right)$ $=\left( \cdots\right)dt+\frac{\partial S}{\partial x}\left( t,x\left( t \right),y\left( t \right) \right)\sigma\left( t \right)dW_{1}\left( t \right)+\frac{\partial S}{\partial y}\left( t,x\left( t \right),y\left( t \right) \right)\eta\left( t \right)dW_{2}\left( t \right)$ $=\sqrt{\left[ \frac{\partial S}{\partial x}\left( \cdot\right)\sigma\left( t \right) \right]^{2}+\left[ \frac{\partial S}{\partial y}\left( \cdot\right)\eta\left( t \right) \right]^{2}+2\rho\sigma\left( t \right)\eta\left( t \right)\frac{\partial S}{\partial x}\left( \cdot\right)\frac{\partial S}{\partial y}\left( \cdot\right)}\cdot dW^{A}\left( t \right)$ $=\sqrt{\left( q_{1}\left( t,x,y \right)\sigma\left( t \right) \right)^{2}+\left( q_{2}\left( t,x,y \right)\eta\left( t \right) \right)^{2}+2\rho q_{1}\left( t,x,y \right)q_{2}\left( t,x,y \right)\sigma\left( t \right)\eta\left( t \right)}\cdot dW^{A}\left( t \right)\text{,}$ | (27) |
| --- | --- | --- |

where $W^{A}$ is the $Q^{A}$-Brownian motion and $P\left( t,T_{i} \right)$ is a function of $x$ and $y$.

Evaluating the partial derivatives yields

|  | $q_{1}\left( t,x,y \right)=-\frac{P\left( t,T_{0},x,y \right)G\left( a,t,T_{0} \right)-P\left( t,T_{N},x,y \right)G\left( a,t,T_{N} \right)}{A\left( t,x,y \right)}+\frac{S\left( t,x,y \right)}{A\left( t,x,y \right)}\sum_{i=0}^{N-1} \tau_{i}P\left( t,T_{i+1},x,y \right)G\left( a,t,T_{i+1} \right)\text{,}$  $q_{2}\left( t,x,y \right)=-\frac{P\left( t,T_{0},x,y \right)G\left( b,t,T_{0} \right)-P\left( t,T_{N},x,y \right)G\left( b,t,T_{N} \right)}{A\left( t,x,y \right)}+\frac{S\left( t,x,y \right)}{A\left( t,x,y \right)}\sum_{i=0}^{N-1} \tau_{i}P\left( t,T_{i+1},x,y \right)G\left( b,t,T_{i+1} \right)\text{,}$ | (28) |
| --- | --- | --- |

where

$$G\left( c,t,T \right)=\frac{1-e^{-c\left( T-t \right)}}{c}\text{.}$$

The function $q_{i}\left( t,x,y \right)$ can be experimentally verified to be close to a constant in the $x$-direction and $y$-direction; thus, as a good approximation, we can write

|  | $q_{i}\left( t,x\left( t \right),y\left( t \right) \right)\approx q_{i}\left( t,\overline{x}\left( t \right),\overline{y}\left( t \right) \right)\text{,}$ | (29) |
| --- | --- | --- |

where $\overline{x}\left( t \right)$ and $\overline{y}\left( t \right)$ are deterministic proxies for $x\left( t \right)$ and $y\left( t \right)$ respectively.

Thus, the option formula is the normal model:

|  | $V_{swaption}\left( 0 \right)\approx A\left( 0 \right)\left[ \left( S\left( 0 \right)-c \right)\Phi\left( d \right)+\sqrt{\nu}\varphi\left( d \right) \right]\text{,}$ | (30) |
| --- | --- | --- |

where

$$d=\frac{S\left( 0 \right)-c}{\sqrt{\nu}}\text{,}$$

$$\nu=\int_{0}^{T_{0}} \left[ \left( q_{1}\left( t,\overline{x}\left( t \right),\overline{y}\left( t \right) \right)\sigma\left( t \right) \right)^{2}+\left( q_{2}\left( t,\overline{x}\left( t \right),\overline{y}\left( t \right) \right)\eta\left( t \right) \right)^{2}+2\rho q_{1}\left( t,\overline{x}\left( t \right),\overline{y}\left( t \right) \right)q_{2}\left( t,\overline{x}\left( t \right),\overline{y}\left( t \right) \right)\sigma\left( t \right)\eta\left( t \right) \right]dt\text{.}$$

Using the freezing technique, assume $\overline{x}\left( t \right)=0$, $\overline{y}\left( t \right)=0$ and $P\left( t,T,x,y \right)=P\left( 0,T \right)$. Then,

|  | $q_{1}\left( t,0,0 \right)=-\frac{P\left( 0,T_{0} \right)G\left( t,T_{0} \right)-P\left( 0,T_{N} \right)G\left( a,t,T_{N} \right)}{A\left( 0 \right)}+\frac{S\left( 0 \right)}{A\left( 0 \right)}\sum_{i=0}^{N-1} \tau_{i}P\left( T_{i+1} \right)G\left( a,t,T_{i+1} \right)$ $=-\frac{1}{A\left( 0 \right)}\left[ \left( \frac{1}{a}-\frac{1}{a}e^{-aT_{0}}e^{a_{t}} \right)P\left( 0,T_{0} \right)-\left( \frac{1}{a}-\frac{1}{a}e^{-aT_{N}}e^{at} \right)P\left( 0,T_{N} \right) \right]+\frac{S\left( 0 \right)}{A\left( 0 \right)}\sum_{i=0}^{N-1} \tau_{i}P\left( 0,T_{i+1} \right)\left( \frac{1}{a}-\frac{1}{a}e^{-aT_{i+1}}e^{at} \right)$ $=\frac{e^{at}}{aA\left( 0 \right)}\left( e^{-aT_{0}}P\left( 0,T_{0} \right)-e^{-aT_{N}}P\left( 0,T_{N} \right) \right)-\frac{1}{aA\left( 0 \right)}\left( P\left( 0,T_{0} \right)-P\left( 0,T_{N} \right) \right) -\frac{e^{at}}{aA\left( 0 \right)}S\left( 0 \right)\sum_{i=0}^{N-1} \tau_{i}P\left( 0,T_{i+1} \right)e^{-aT_{i+1}}+\frac{1}{aA\left( 0 \right)}S\left( 0 \right)\sum_{i=0}^{N-1} \tau_{i}P\left( 0,T_{i+1} \right)$ $=e^{at}\times\frac{e^{-aT_{0}}P\left( 0,T_{0} \right)-e^{-aT_{N}}P\left( 0,T_{N} \right)-S\left( 0 \right)\sum_{i=0}^{N-1} \tau_{i}P\left( 0,T_{i+1} \right)e^{-aT_{i+1}}}{aA\left( 0 \right)}$ $=e^{at}\times C_{1,T_{0},T_{N}}=\tilde{q}_{1}\left( t,T_{0},T_{N} \right)\text{.}$ | (31) |
| --- | --- | --- |

In the case of $q_{2}\left( t,0,0 \right)$, $a$ is replaced by $b$.

Therefore, we obtain the variance of swap rate:

|  | $\nu=\int_{0}^{T_{0}} \left[ \tilde{q}_{1}\left( t,T_{0},T_{N} \right)^{2}\sigma\left( t \right)^{2}+\tilde{q}_{2}\left( t,T_{0},T_{N} \right)^{2}\eta\left( t \right)^{2}+2\tilde{q}_{1}\left( t,T_{0},T_{N} \right)\tilde{q}_{2}\left( t,T_{0},T_{N} \right)\rho\sigma\left( t \right)\eta\left( t \right) \right]dt$ $=C_{1,T_{0},T_{N}}^{2}\int_{0}^{T_{0}} e^{2at}\sigma\left( t \right)^{2}dt+C_{2,T_{0},T_{N}}^{2}\int_{0}^{T_{0}} e^{2bt}\eta\left( t \right)^{2}dt+2{\rho C}_{1,T_{0},T_{N}}C_{2,T_{0},T_{N}}\int_{0}^{T_{0}} e^{\left( a+b \right)t}\sigma\left( t \right)\eta\left( t \right)dt\text{.}$ | (32) |
| --- | --- | --- |

If $\sigma\left( t \right)$ and $\eta\left( t \right)$ are piecewise constant,

|  | $\nu\left( T_{0},T_{N} \right)=\frac{C_{1,T_{0},T_{N}}^{2}}{2a}\sum_{j=k}^{m} \sigma_{j+1}^{2}\left( e^{2aT_{j+1}}-e^{2aT_{j}} \right)+\frac{C_{2,T_{0},T_{N}}^{2}}{2b}\sum_{j=k}^{m} \eta_{j+1}^{2}\left( e^{2bT_{j+1}}-e^{2bT_{j}} \right)+2\frac{C_{1,T_{0},T_{N}}C_{2,T_{0},T_{N}}}{\left( a+b \right)}\sum_{j=k}^{m} \rho\sigma_{j+1}\eta_{j+1}\left( e^{\left( a+b \right)T_{j+1}}-e^{\left( a+b \right)T_{j}} \right)\text{.}$ | (33) |
| --- | --- | --- |

Because we assume that $\sigma\left( t \right)$ and $\eta\left( t \right)$ are constant, $\sigma$ and $\eta$ respectively,

|  | $\nu\left( T_{0},T_{N} \right)=\frac{C_{1,T_{0},T_{N}}^{2}}{2a}\sigma^{2}\left( e^{2aT_{N}}-e^{2aT_{0}} \right)+\frac{C_{2,T_{0},T_{N}}^{2}}{2b}\eta^{2\left( e^{2bT_{N}}-e^{2bT_{0}} \right)}+2\frac{C_{1,T_{0},T_{N}}C_{2,T_{0},T_{N}}}{\left( a+b \right)}\rho\sigma\eta\left( e^{\left( a+b \right)T_{N}}-e^{\left( a+b \right)T_{0}} \right)\text{.}$ | (34) |
| --- | --- | --- |
