# Supplementary material for: How to calibrate Gaussian two-factor model using swaption
Source: PLoS One. 2023 Feb 23;18(2):e0280829. doi: 10.1371/journal.pone.0280829 (PMC9949672; doi:10.1371/journal.pone.0280829)

**S2 Appendix: Other numerical results (USD Currency)**

**S2 Table 1. Calibration basket for step (1) of the two-step approach in USD currency.**

This table presents the calibration basket for step (1) of the two-step approach in the USD currency. Based on volatility with a swap maturity of 20Y, the basket comprises the swaption volatility ratio with swap maturity 1Y, the shortest swap maturity on the market. In our test case, the non-call period is 1-year; thus, we use option expiry from 1Y to 30Y. Further, we include the ratio of volatility with a swap maturity of 10Y in the basket.

| **Option Maturity** | **Swap Maturity in denominator** | **Swap Maturity in numerator** | **Swaption Normal Volatility Ratio** |
| --- | --- | --- | --- |
| 1Y | 20Y | 1Y | $\mathcal{V}^{mkt}\left( 1Y,20Y \right)/\mathcal{V}^{mkt}\left( 1Y,1Y \right)$ |
|  |  | 10Y | $\mathcal{V}^{mkt}\left( 1Y,20Y \right)/\mathcal{V}^{mkt}\left( 1Y,10Y \right)$ |
| 2Y | 20Y | 1Y | $\mathcal{V}^{mkt}\left( 2Y,20Y \right)/\mathcal{V}^{mkt}\left( 2Y,1Y \right)$ |
|  |  | 10Y | $\mathcal{V}^{mkt}\left( 2Y,20Y \right)/\mathcal{V}^{mkt}\left( 2Y,10Y \right)$ |
| 3Y | 20Y | 1Y | $\mathcal{V}^{mkt}\left( 3Y,20Y \right)/\mathcal{V}^{mkt}\left( 3Y,1Y \right)$ |
|  |  | 10Y | $\mathcal{V}^{mkt}\left( 3Y,20Y \right)/\mathcal{V}^{mkt}\left( 3Y,10Y \right)$ |
| 4Y | 20Y | 1Y | $\mathcal{V}^{mkt}\left( 4Y,20Y \right)/\mathcal{V}^{mkt}\left( 4Y,1Y \right)$ |
|  |  | 10Y | $\mathcal{V}^{mkt}\left( 4Y,20Y \right)/\mathcal{V}^{mkt}\left( 4Y,10Y \right)$ |
| 5Y | 20Y | 1Y | $\mathcal{V}^{mkt}\left( 5Y,20Y \right)/\mathcal{V}^{mkt}\left( 5Y,1Y \right)$ |
|  |  | 10Y | $\mathcal{V}^{mkt}\left( 5Y,20Y \right)/\mathcal{V}^{mkt}\left( 5Y,10Y \right)$ |
| 7Y | 20Y | 1Y | $\mathcal{V}^{mkt}\left( 7Y,20Y \right)/\mathcal{V}^{mkt}\left( 7Y,1Y \right)$ |
|  |  | 10Y | $\mathcal{V}^{mkt}\left( 7Y,20Y \right)/\mathcal{V}^{mkt}\left( 7Y,10Y \right)$ |
| 10Y | 20Y | 1Y | $\mathcal{V}^{mkt}\left( 10Y,20Y \right)/\mathcal{V}^{mkt}\left( 10Y,1Y \right)$ |
|  |  | 10Y | $\mathcal{V}^{mkt}\left( 10Y,20Y \right)/\mathcal{V}^{mkt}\left( 10Y,10Y \right)$ |
| 15Y | 20Y | 1Y | $\mathcal{V}^{mkt}\left( 15Y,20Y \right)/\mathcal{V}^{mkt}\left( 15Y,1Y \right)$ |
|  |  | 10Y | $\mathcal{V}^{mkt}\left( 15Y,20Y \right)/\mathcal{V}^{mkt}\left( 15Y,10Y \right)$ |
| 20Y | 20Y | 1Y | $\mathcal{V}^{mkt}\left( 20Y,20Y \right)/\mathcal{V}^{mkt}\left( 20Y,1Y \right)$ |
|  |  | 10Y | $\mathcal{V}^{mkt}\left( 20Y,20Y \right)/\mathcal{V}^{mkt}\left( 20Y,10Y \right)$ |
| 25Y | 20Y | 1Y | $\mathcal{V}^{mkt}\left( 25Y,20Y \right)/\mathcal{V}^{mkt}\left( 25Y,1Y \right)$ |
|  |  | 10Y | $\mathcal{V}^{mkt}\left( 25Y,20Y \right)/\mathcal{V}^{mkt}\left( 25Y,10Y \right)$ |
| 30Y | 20Y | 1Y | $\mathcal{V}^{mkt}\left( 30Y,20Y \right)/\mathcal{V}^{mkt}\left( 30Y,1Y \right)$ |
|  |  | 10Y | $\mathcal{V}^{mkt}\left( 30Y,20Y \right)/\mathcal{V}^{mkt}\left( 30Y,10Y \right)$ |

**S2 Fig1**. **(Method I) Model parameters calibrated to 20Y co-terminal and 10Y-2Y spread with** $\boldsymbol{\rho}\mathbf{=-0.9}$**.**

Panel A shows the mean reversions calibrated with step (1) of the two-step approach, and Panel B shows the volatility parameters calibrated with step (2). A significant change existed in the 2nd quarter of 2019 in all panels. This is because the swap rates declined owing to cuts in the US and Korea’s benchmark rate, and the swaption normal volatility rose.


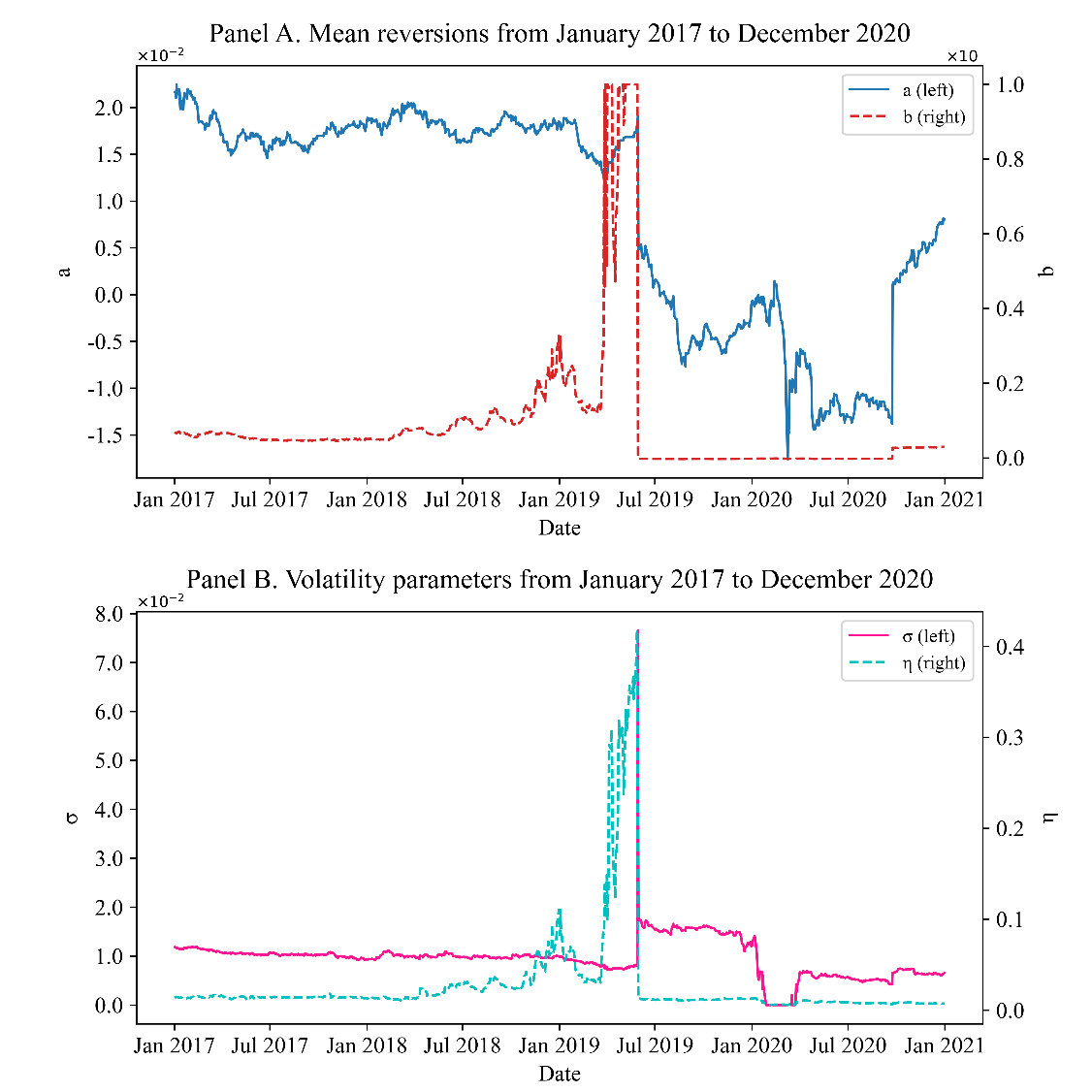


**S2 Fig2**. **(Method II) Model parameters calibrated to 20Y co-terminal and 10Y-2Y spread with** $\boldsymbol{\rho}\mathbf{=-0.9}$**.**

Panels A and B show the mean reversions and volatility parameters, respectively, calibrated with the one-step method. The mean reversions and volatility parameters change significantly every day. Many significant changes occur (e.g., the 2nd and 3rd quarters of 2019).


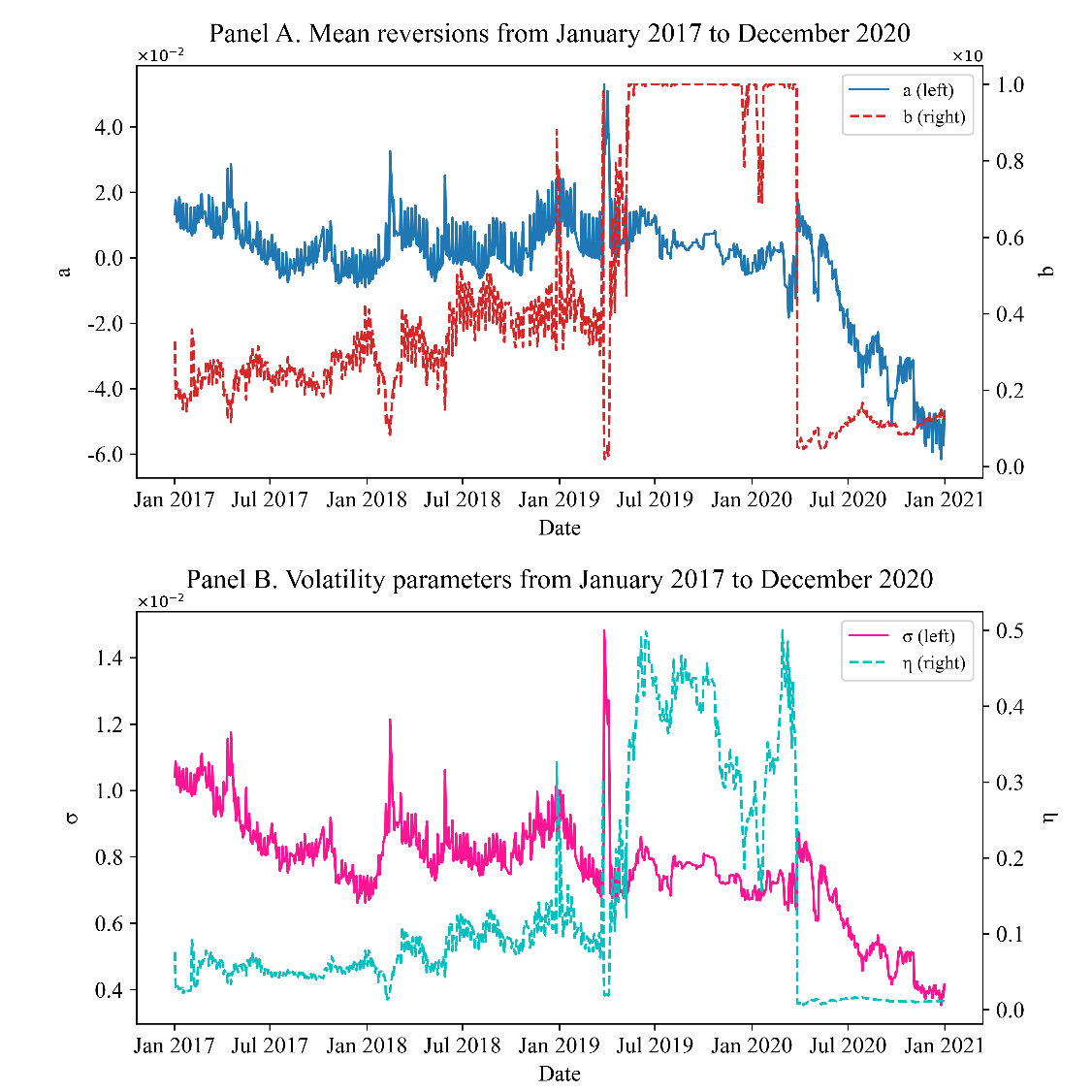


**S2 Fig3**. **(Method III) Model parameters (include** $\boldsymbol{\rho}$**) calibrated to 20Y co-terminal and 10Y-2Y spread.**

Panels A, B, and C show the mean reversions, volatility parameters, and correlation coefficient, respectively, calibrated with the one-step method. Here, the correlation coefficient stands out and is sensitive to changes in market data. Whereas the mean reversion rarely moves, the others change significantly. However, the volatility parameters move in the same direction.


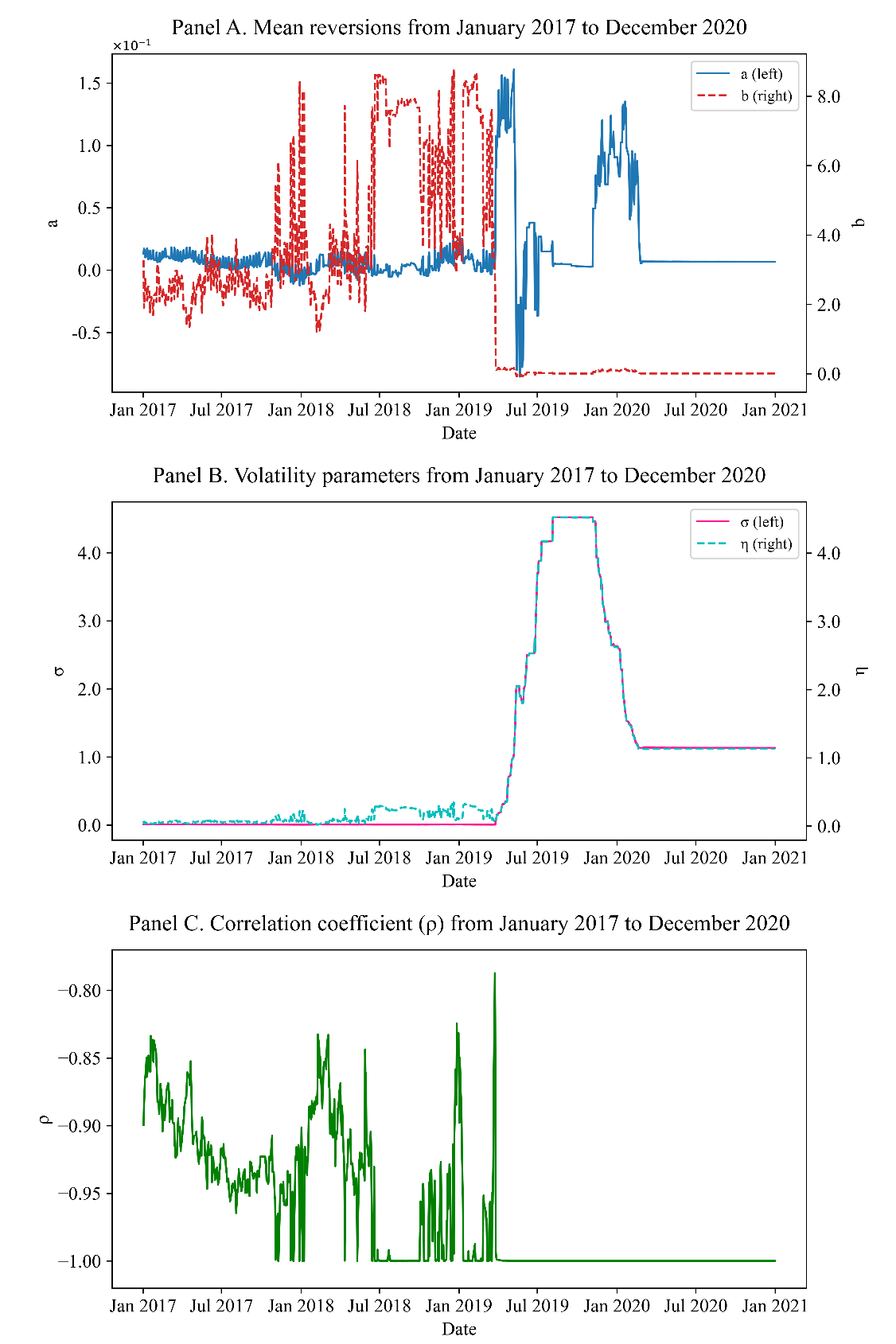

Supplement: S2 Appendix — (DOCX) [file pone.0280829.s002.docx]
